# Temporal and spatial profile of polymorphonuclear myeloid-derived suppressor cells (PMN-MDSCs) in ischemic stroke in mice

**DOI:** 10.1371/journal.pone.0215482

**Published:** 2019-05-02

**Authors:** Tomohiro Kawano, Munehisa Shimamura, Hironori Nakagami, Hideaki Kanki, Tsutomu Sasaki, Hideki Mochizuki

**Affiliations:** 1 Department of Neurology, Osaka University Graduate school of Medicine, Suita, Osaka, Japan; 2 Department of Health Development and Medicine, Osaka University Graduate School of Medicine, Suita, Osaka, Japan; Ehime University Graduate School of Medicine, JAPAN

## Abstract

Although T cells play important roles in the pathophysiology of ischemic stroke, the dynamics of T cells remains unclear. In cancer, polymorphonuclear myeloid-derived suppressor cells (PMN-MDSCs) contribute to the maintenance of the tumor microenvironment by suppressing T cells. However, the presence of these cells has never been examined in ischemic brain. Therefore, we examined the temporal and spatial profiles of PMN-MDSCs, which are defined as the CD11b^+^Ly6C^low^Ly6G^+^ cells with higher expression levels of *Nox2* and *C/EBP Homologous Protein (CHOP)* mRNA than normal neutrophil. Fluorescence-activated cell sorter (FACS) analysis showed that the count of CD11b^+^Ly6C^low^Ly6G^+^ cells was increased in the ischemic hemisphere and bone marrow at 72 hours, as well as in the spleen 24 hours after transient middle cerebral artery occlusion in mice. In contrast, the contralateral hemisphere, normal bone marrow, and normal spleen contained few CD11b^+^Ly6C^low^Ly6G^+^ cells. Real-time reverse transcription polymerase chain reaction revealed that CD11b^+^Ly6C^low^Ly6G^+^ cells sorted from brain and spleen 72 hours after ischemia had greater expression of *Nox2* and *CHOP* mRNA than neutrophils in bone marrow, suggesting that these cells constitute PMN-MDSCs. Immunohistochemistry showed that CD11b^+^Ly6G^+^ cells were located in the ischemic core and border zone, indicating that PMN-MDSCs might be endemic to these regions. Although neutrophils are believed to invade infarct regions 48–72 hours after ischemia, the present study suggested that some of these cells are in fact PMN-MDSCs. Further studies on the function of PMN-MDSCs might unveil the unknown mechanisms of T cell activation and recruitment in ischemic stroke.

## Introduction

Inflammation and immune cells play important roles in the pathology of ischemic stroke. Following brain ischemia, microglia are activated by damage-associated molecular patterns (DAMPs), such as reactive oxygen species (ROS) and high-mobility group box 1, which are released from injured cells [1]. Activated microglia release several proinflammatory cytokines, such as interleukin 1-beta (IL-1β), IL-6, and tumor necrosis factor (TNF), which prime dendritic cells for antigen presentation. Activated dendritic cells then facilitate T cell responses [1].

However, in patients with cancer, myeloid-derived suppressor cells (MDSCs) inhibit T cell responses. MDSCs support tumor growth, differentiation, and metastasis by inhibiting T cell activation and proliferation [2]. They comprise a heterogeneous population of immature myeloid cells that can be divided into two major subsets based on phenotype and morphology: polymorphonuclear (PMN-) and monocytic (M)-MDSCs [3]. In mice, the surface marker of PMN-MDSCs is CD11b^+^Ly6C^low^Ly6G^+^, while that of M-MDSCs is CD11b^+^Ly6C^hi^Ly6G^-^ [3–5]. The ability to suppress T cell activity distinguishes PMN-MDSCs from neutrophils, which have identical cell surface markers. Therefore, functional assays or biochemical and molecular parameters associated with T cell suppression are necessary to identify PMN-MDSCs. Such parameters are *Nox2* and *C/EBP Homologous Protein (CHOP)* mRNA expression, which is higher in PMN-MDSCs than in neutrophils in normal bone marrow [3, 6, 7].

Furthermore, the mechanisms of immune suppression differ somewhat between PMN-MDSCs and M-MDSCs. PMN-MDSCs increase the activity of the signal transducer and activator of transcription 3 (STAT3) and nicotinamide adenine dinucleotide phosphate, resulting in high levels of ROS but low nitric oxide (NO) production. ROS and peroxynitrite induce post-translational modification of T cell receptors and may cause antigen-specific T cell unresponsiveness. In contrast, M-MDSCs upregulate the expression of STAT1 and inducible nitric oxide synthase (iNOS), leading to increased levels of NO but low ROS production. NO suppresses T cell function by inhibiting Janus kinase 3 and STAT5, preventing histocompatibility complex class II expression and leading to T cell apoptosis [8]. Thus, ROS production, which is indicated by increased expression of *Nox2* mRNA, is a characteristic feature of PMN-MDSCs [6, 9]. Although M-MDSCs are more potent suppressors of T cell response on a per cell basis [2, 10], PMN-MDSC depletion leads to greater improvements in antitumor immunity [11, 12] than M-MDSC depletion [13]. Thus, it is more important to regulate PMN-MDSC population than M-MDSC count in the treatment of tumors. Additionally, PMN-MDSCs suppress immune responses in chronic infectious disease, trauma, sepsis, and many other pathological conditions [14]. These indicate that PMN-MDSCs may be more potent than M-MDSCs in regulating immune responses in ischemic stroke. Thus, we focused on PMN-MDSCs in the present study.

To clarify whether PMN-MDSCs are involved in the pathophysiology of the ischemic brain, we examined the temporal and spatial profile of CD11b^+^Ly6C^low^Ly6G^+^ cells and *Nox2* and *CHOP* mRNA expression using the transient focal ischemic model.

## Materials and methods

### Mice

This study was fully approved by the Ethics Committee for Animal Experiments of Osaka University Graduate School of Medicine. Seven-week-old male C57BL/6J mice were purchased from CLEA Japan, Inc. and housed in a temperature- and light cycle-controlled animal facility with free access to food and water. 118 mice were included in the present study.

### Transient focal cerebral ischemia

Transient middle cerebral artery occlusion (MCAo) was performed as previously described [15]. Briefly, the mice were anesthetized using isoflurane (1.4%), and their cerebral blood flow was measured using a laser Doppler flowmeter (Unique Acquisition software; Unique Medical). A 6.0 monofilament surgical suture was inserted into the external carotid artery and advanced into the internal carotid artery to obstruct the origin of the middle cerebral artery. The filament was left in place for 30 minutes and then withdrawn. In the present study, we only included animals that (1) exhibited an immediate reduction of cerebral blood flow (CBF) after MCAo with subsequent cycles of slight increase and drop of CBF [16], (2) showed a more than 82% reduction in CBF with and (3) displayed a 30%–80% recovery in CBF within 5 minutes of reperfusion. The rectal temperature of all mice was maintained at 37.0 ± 0.5 °C during surgery. As controls, age-matched naïve mice that were not subjected to MCAo were used.

### Brain immune cell isolation

As described in detail elsewhere [17], mice were deeply anesthetized using a combination anesthetic consisting of 0.3 mg/kg of medetomidine, 4.0 mg/kg of midazolam, and 5.0 mg/kg of butorphanol by intraperitoneal injection. They were then transcardially perfused using 20 mL of ice-cold HBSS (Wako, Osaka, Japan). Their brains were removed and separated into left (contralateral) and right (ischemic) hemispheres. Each hemisphere was dissociated mechanically in cold RPMI 1640 medium (RPMI; GIBCO, Grand Island, NY, USA). Mouse brain immune cells were obtained using the Percoll density gradient method (GE Healthcare, Pittsburgh, PA, USA) and centrifuged at 500 × *g* for 30 continuous minutes at 18°C. The interphase (3 mL) was then collected and centrifuged at 500 × *g* for 7 minutes at 18°C. The pellet was resuspended in 1 mL of fetal bovine serum (BD Pharmingen) on ice; the solution was then centrifuged at 9700 × *g* for 1 minute at 4°C. The washed cells were resuspended in 200 μl of fetal bovine serum and analyzed immediately in the fluorescence-activated cell sorter (FACS).

### Spleen and bone marrow cell isolation

Spleens were mechanically dissociated and passed through 40-μm nylon cell strainers (BD Falcon 352340; BD Biosciences, San Jose, CA, USA) to obtain a single-cell suspension. Bone marrow cells were flushed from femurs and tibiae using RPMI medium; they were then passed through the same 40-μm nylon cell strainers. These cells were then lysed using red blood cell lysis buffer (BD Biosciences), washed, and resuspended in isolation buffer at a concentration of 1 × 10^7^ cells /mL.

### Fluorescence-activated cell sorter analysis and cell sorting

The cells were stained with APC-Cy7-conjugated anti-CD45 (BD Pharmingen), PE-conjugated anti-CD11b (BD Pharmingen), APC-conjugated anti-Ly6G (BD Pharmingen), and BV421-conjugated anti-Ly6C (BD Horizon). Dead cells and debris were gated out using forward light scatter, side light scatter, and 7-AAD (BD Biosciences, San Jose, CA). FACS analysis was performed on a FACSCanto II flow cytometer and analyzed using FACS Diva software (BD Biosciences). Cell sorting was performed using FACSAria II (BD Biosciences).

### Real-time reverse transcription polymerase chain reaction

Seventy-two hours after MCAo, the ischemic hemisphere, contralateral hemisphere and spleen were collected, as was normal bone marrow. The FACS-sorted CD11b^+^Ly6C^low^Ly6G^+^ cells were lysed using the cells-to-CT1 Step TaqMan Kit (Thermo Fischer Scientific), according to the manufacturer’s recommendations. The resulting lysate was then used in a one-step, real-time reverse transcription polymerase chain reaction (RT-PCR). Specifically, TaqMan gene expression assay for Nox2 (*Cybb*, assay No. Mm01287743_m1), CHOP (*Ddit3*, assay No. Mm00492097_m1), and Nox4 *(Nox4*, assay No. Mm00479246_m1) were used, while glyceraldehyde 3-phosphate dehydrogenase (*GAPDH*) was used as an endogenous reference (assay Mm99999915_g1). The RT-PCR used a 7900HT fast real-time PCR system (Applied Biosystems, Carlsbad, CA, USA). Expression was assessed in triplicate. Relative quantification method 2-[Delta][Delta] Ct (2^-deltalelta Ct^) was used for normalization of gene expression [18]. All Ct values beyond 40-cycle was excluded from data analysis. Gene expression was normalized with the *GAPDH*. The level of the gene expression of the ischemic hemisphere, contralateral hemisphere, and spleen was compared with the level of the gene expression in normal bone marrow and expressed as an n-fold ratio.

### Immunohistochemical staining

Seventy-two hours after MCAo, the mice were perfused with 4% paraformaldehyde, and their brains were cut into slices 12 μm in thickness. Frozen sections were washed twice in PBS for 5 minutes and then rinsed for 30 minutes in PBS with 0.3% hydrogen peroxide (Wako), ensuring that any endogenous peroxidase was inactivated. After this peroxidase treatment, the sections were washed twice in PBS for 5 minutes and then incubated for 30 minutes in PBS with 20% normal goat serum (Vector laboratories, Burlingame, CA, USA). The sections were then incubated for 60 minutes at room temperature in anti-Ly6G antibody (1:50; rat anti-mouse Ly6G clone 1A8; BD Pharmingen) dissolved in PBS with 20% goat serum. As negative controls, normal control IgG (1:50; Santa Cruz Biotechnology, Dallas, Texas, USA) was applied instead of the anti-Ly6G antibody. The sections were incubated for 30 minutes at room temperature in goat anti-rat IgG biotinylated antibody (1:200; Vector; dissolved in PBS with 20% goat serum). The sections were incubated for 30 minutes at room temperature in horse radish peroxidase-conjugated streptavidin (1:100; PerkinElmer, Inc. Waltham, MA, USA; dissolved in PBS with 20% goat serum). The sections were incubated for 10 minutes at room temperature in cyanine-5 amplification reagent (1:50; PerkinElmer). The sections were incubated for 60 minutes at room temperature in FITC-conjugated anti-CD11b antibody (1:100; ab24874; Abcam, Cambridge, UK; dissolved in DAKO REAL antibody diluent (DAKO, Glostrup, Denmark). The sections were mounted using VECTASHIELD mounting medium (Vector Laboratories). After every step, the samples were rinsed three times in PBS. The images were examined using a confocal microscope (FV10i; Olympus, Tokyo, Japan). We visually assessed and counted the number of Merged cells.

### Statistics

All values are expressed as mean ± standard deviation. The nonparametric Kruskal–Wallis test followed by Dunn’s post hoc test were used to test multiple comparison between groups. Statistical analyses were performed using GraphPad Prism software version 6.07 (GraphPad Inc., San Diego, CA, USA). P-values less than 0.05 were considered statistically significant.

## Results

### Increased CD11b^+^Ly6C^low^Ly6G^+^ cells in ischemic brain, bone marrow and spleen

**Fig 1:**
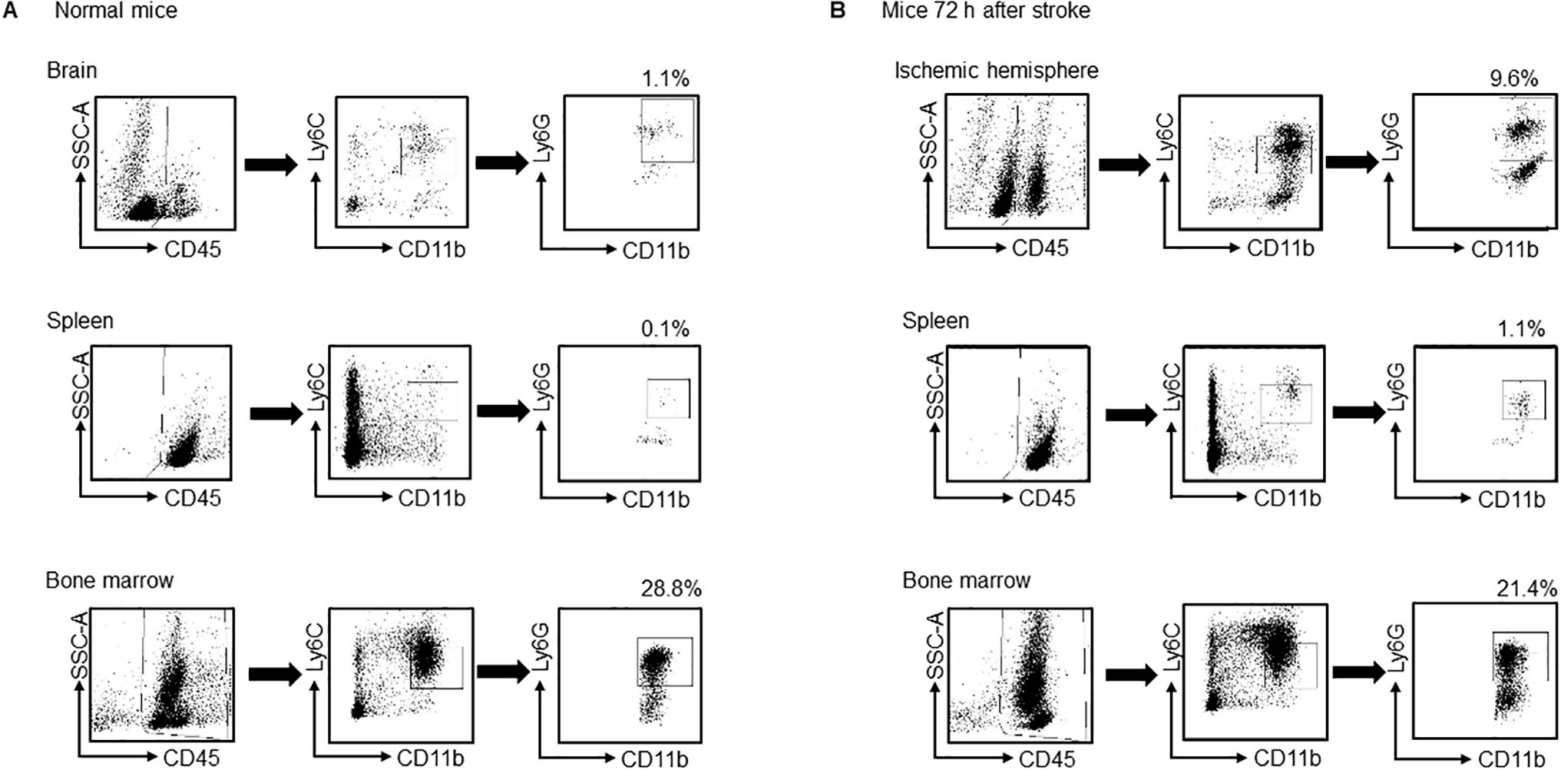
Gating strategy to identify CD11b^+^Ly6C^low^Ly6G^+^ cells in brain, spleen, and bone marrow. The gating strategy used to identify PMN-MDSCs subpopulations in brain, spleen, and bone marrow in (A) normal mice and (B) mice 72 hours after stroke (MCAo). After exclusion of duplicates, live CD45^+^ cells were gated, and the proportion of CD11b^+^Ly6C^low^Ly6G^+^ cells was quantified and shown as a percentage (%).

**Fig 2:**
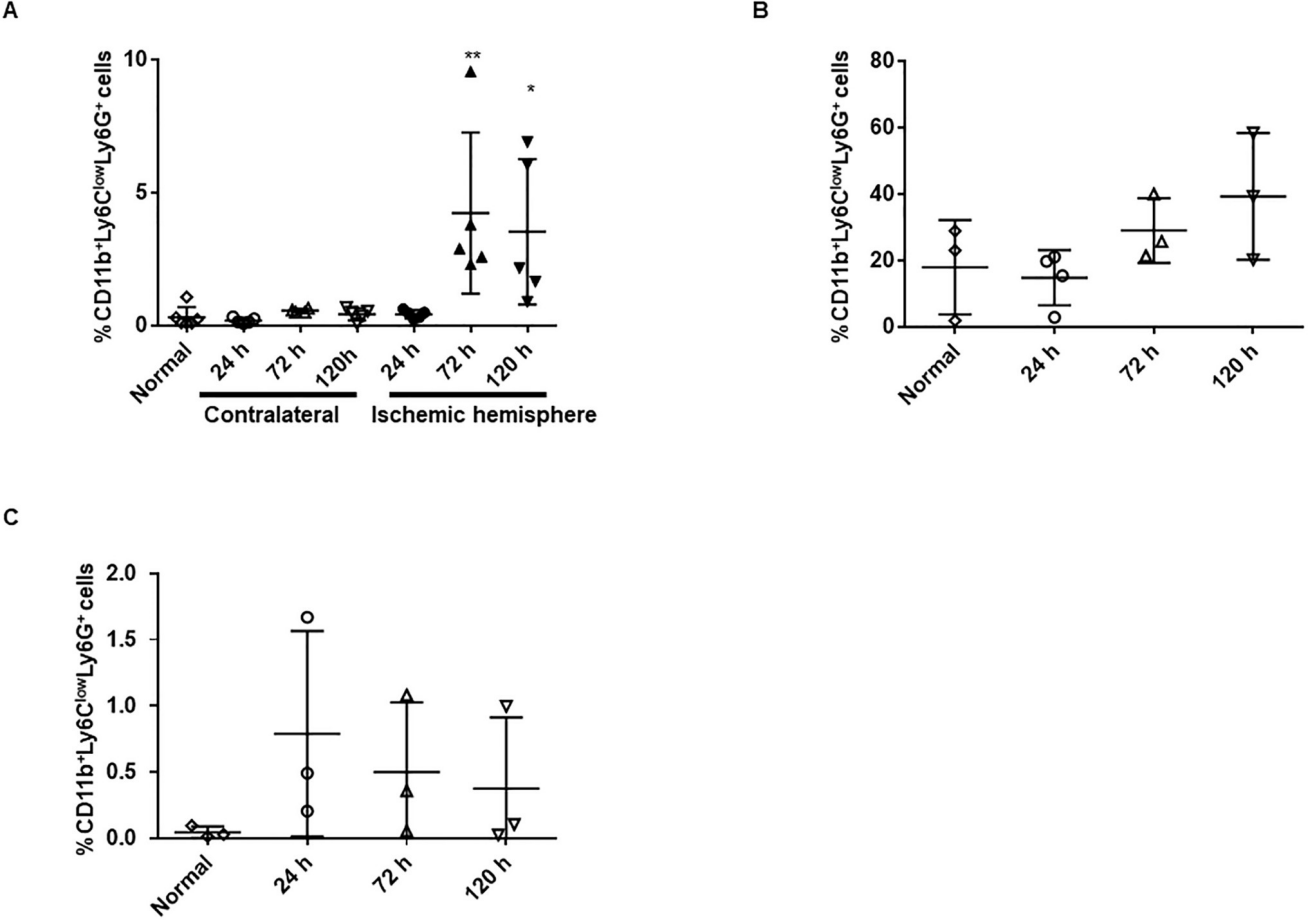
Temporal profile of CD11b^+^Ly6C^low^Ly6G^+^ cells in brain, bone marrow, and spleen. The number of CD11b^+^Ly6C^low^Ly6G^+^ cells (gated on live CD45^+^ cells) was evaluated in (A) brain (ischemic and contralateral hemispheres; n = 4–6, respectively), (B) spleen (n = 3), and (C) bone marrow (n = 3–4, respectively) at 24 hours, 48 hours, and 120 hours after MCAo. Values are shown as mean ± SD.*p < 0.05, **p < 0.01 vs. normal group.

Because the cell marker of PMN-MDSCs is defined as CD11b^+^Ly6C^low^Ly6G^+^, we first examined the temporal profile of CD11b^+^Ly6C^low^Ly6G^+^ cells in the brain, spleen, and bone marrow after ischemic stroke using FACS analysis (Fig 1). In brain, the count of CD11b^+^Ly6C^low^Ly6G^+^ cells was significantly increased at both 72 hours and 120 hours after stroke in the ischemic hemisphere, whereas it remained unchanged in the contralateral hemisphere (Fig 2A). Correspondingly, the number of CD11b^+^Ly6C^low^Ly6G^+^ cells in the bone marrow had not increased 24 hours after ischemia, but there was a tendency towards increase at 72 hours and 120 hours after ischemia (Fig 2B). In contrast, the number of CD11b^+^Ly6C^low^Ly6G^+^ cells in spleen was increased 24 hours after stroke, followed by a gradual decrease (Fig 2C). These data indicated that CD11b^+^Ly6C^low^Ly6G^+^ cells were increased in spleen and subsequently in both ischemic brain and bone marrow.

### CD11b^+^Ly6C^low^Ly6G^+^ cells in brain and spleen after MCAo includes PMN-MDSC-like cells

**Fig 3:**
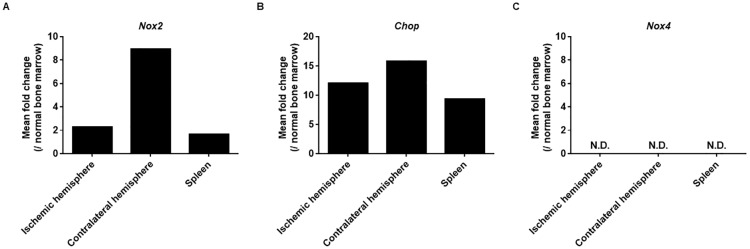
CD11b^+^Ly6C^low^Ly6G^+^ cells in the brain and spleen 72 hours after MCAo showed increased expression of *Nox2* and *CHOP* than such cells in the bone marrow of naïve mice. CD11b^+^Ly6C^low^Ly6G^+^ cells were collected from the bone marrow of normal mice, as well as from the spleen, ischemic hemisphere, and contralateral hemisphere 72 hours after MCAo. Because few CD11b^+^Ly6C^low^Ly6G^+^ cells were found in each sample, 5–6 samples were pooled in each group and mRNA was purified from the pooled sample. These analyses were conducted by two independent groups of different samples. Values are shown as mean. N.D.: not detected.

Based on the most recent method of PMN-MDSC identification [3], we next examined whether CD11b^+^Ly6C^low^Ly6G^+^ cells sorted after MCAo showed higher expression of characteristic mRNA than such cells in normal bone marrow, which are neutrophils [3, 8]. The CD11b^+^Ly6C^low^Ly6G^+^ cells in the brain and spleen 72 hours after MCAo expressed higher levels of *Nox2* and *CHOP* mRNA than such cells in the bone marrow of naïve mice, indicating that CD11b^+^Ly6C^low^Ly6G^+^ cells in the brain and spleen are PMN-MDSC-like cells (PMN-MDSC-LCs) [3, 6, 7] rather than neutrophils (Fig 3A and 3B). We also examined the level of *Nox4* mRNA, but it was not detected (Fig 3C).

### Spatial profiles of CD11b^+^Ly6G^+^ cells in the ischemic hemisphere 72 hours after stroke

**Fig 4:**
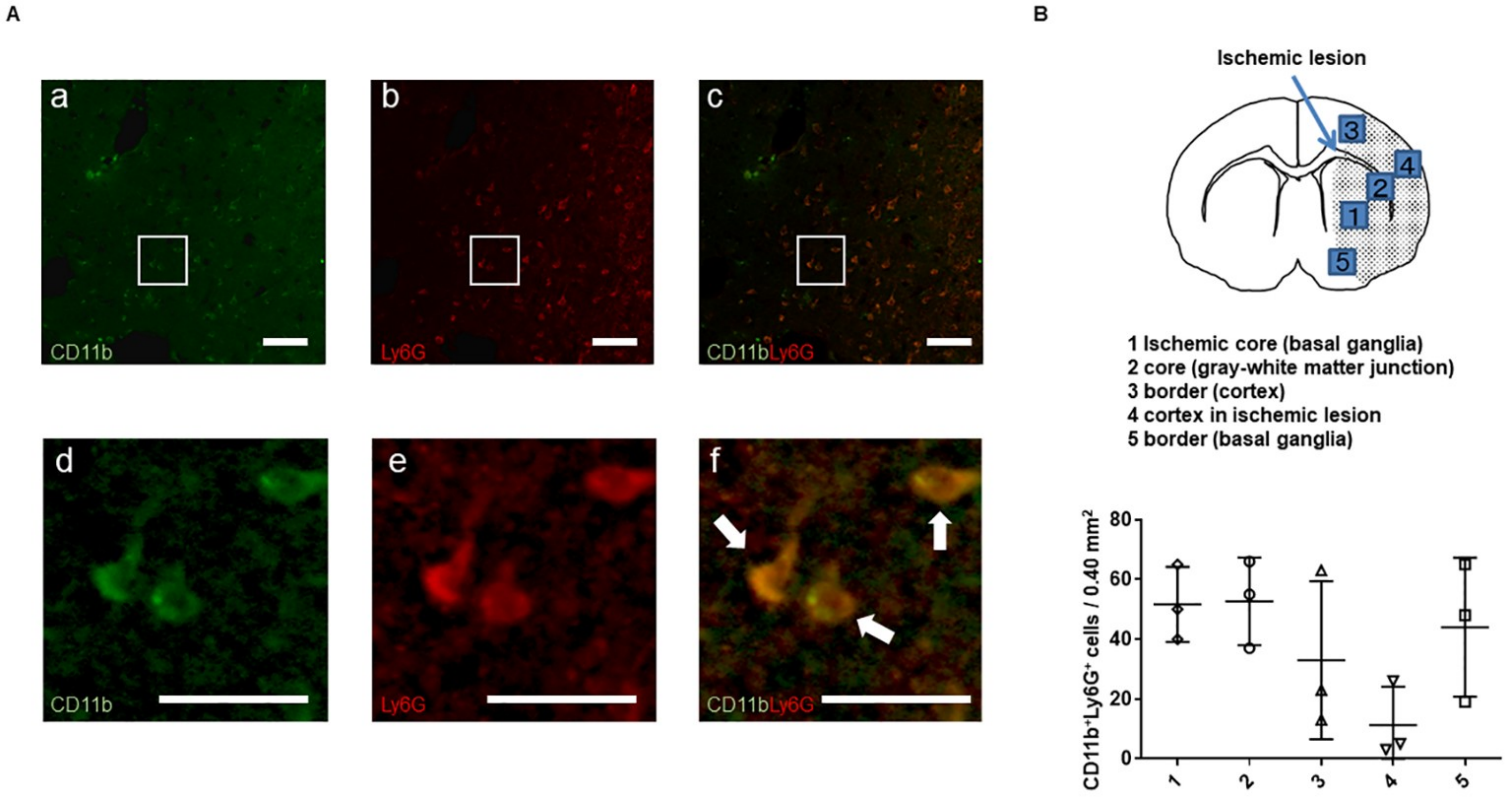
Spatial profile of CD11b^+^Ly6G^+^ cells in ischemic lesions 72 hours after MCAo. (A) Typical images of CD11b^+^Ly6G^+^ cells in the peri-infarct cortex at 72 hours after MCAo (a-f). The sections were double stained for CD11b (green) and Ly6G (red). (f) Arrows indicate double-labeling cells. (d-f) Images in high magnification, which are correspondent to the rectangle regions in a-c. Scale bar: 100 μm (upper panel) and 50 μm (lower panel). (B) CD11b^+^Ly6G^+^ cells were quantified in each lesion in the ischemic hemisphere. The left panel shows the defined ischemic lesion: (1) ischemic core (basal ganglia), (2) core (gray–white matter junction), (3) border of cortex, (4) cortex in ischemic lesion, (5) border of basal ganglia. Values are shown as mean ± SD. n = 3/group.

Next, we examined where PMN-MDSC-LCs occurred in the ischemic hemisphere. Because no clear immunohistochemical, phenotypic characterization of PMN-MDSCs has been published [3], we investigated the distribution of CD11b^+^Ly6G^+^ cells, as in previous reports, defining CD11b^+^Ly6G^+^ cells as PMN-MDSC [19, 20]. Although CD11b^+^Ly6G^+^ cells were observed in the ischemic hemisphere (Fig 4A), quantitative analysis showed that they were observed in the ischemic core (basal ganglia and gray–white matter junction), as well as in the border zone of the basal ganglia (Fig 4B). These results suggested that PMN-MDSC-LCs occur in both the ischemic core and border zone.

## Discussion

In the present study, we demonstrated that CD11b^+^Ly6C^low^Ly6G^+^ cells with high expression of *Nox2* and *CHOP* mRNA were present in the brain and spleen after ischemic stroke. Because the expression pattern of *Nox2* and *CHOP* mRNA is different in neutrophils, these cells must be PMN-MDSC-LCs.

MDSCs are generated in the bone marrow from common myeloid progenitor cells [2], and cancer or acute inflammation markedly increases the number of MDSCs as part of the host immune response [2]. The spleen also acts as an amplifier to promote MDSC proliferation [21], and as a reservoir of MDSCs [21, 22]. In the present study, the count of PMN-MDSCs in the spleen was increased 24 hours after cerebral ischemia, and those in the bone marrow and ischemic brain were increased 72 hours after injury. It follows that cerebral ischemia may quickly induce PMN-MDSC proliferation in the spleen, without migration to the brain, and subsequently accelerate PMN-MDSC increases in bone marrow, with migration to ischemic brain.

In the present study, the expression levels of *Nox2* and *CHOP* mRNA in brain and spleen after ischemic stroke were higher compared to the control neutrophils. Both *Nox2* and *CHOP* are molecular parameters associated with PMN-MDSCs characterization [3]. Interestingly, there was a tendency of high expression of *Nox2* RNA in contralateral hemisphere compared to ischemic hemisphere and spleen, but the expression level of *CHOP* mRNA did not differ. Considering that the production of Nox2 is regulated by transcriptional factors, such as STAT family, but CHOP is modulated by the activating-transcription factor-4, some different factors in different tissues might have affected the *Nox2* gene expression. Because mRNA levels is not necessarily coincident with enzyme activities in MDSC [3], further studies are necessary to clarify the functional differences in PMN-MDSC between the ischemic and contralateral hemisphere after stroke.

Nox family consists of Nox1, Nox2, Nox3, Nox4, Nox5, Duox1, and Duox2 [23]. Their expression is dependent on cells and Nox2 is only molecule reported to be crucial for PMN-MDSCs biology and for their identification [3, 13]. Because we collected CD11b^+^Ly6C^low^Ly6G^+^ cells after gating with CD45, which is the marker for hematopoietic cells except erythrocytes and platelets, we examined the level of *Nox4* mRNA, which is another Nox family expressed in hematopoietic cells, especially in hematopoietic stem cells [23]. As expected, no expression of *Nox4* mRNA was detected in CD11b^+^Ly6C^low^Ly6G^+^ cells. This result also supported our findings that CD11b^+^Ly6C^low^Ly6G^+^ cells included PMN-MDSCs as previously reported [3, 13].

After cerebral ischemia, T cells were found in the peri-infarct area [24], and neutrophils were seen in the overall infarct lesion [25]. The CD11b^+^Ly6G^+^ cells could communicate directly with T cells in the peri-infarct area; these may have been PMN-MDSCs, the immunosuppressive activities of which requires direct cell-cell contact through cell-surface receptors and/or through the release of short-lived soluble mediators [2, 8]. Conversely, the CD11b^+^Ly6G^+^ cells in the ischemic core may have been neutrophils.

How are PMN-MDSCs recruited to the ischemic brain? At the steady state, the population of MDSCs is low both in mice and human. However, MDSCs are accumulated in various diseases, including cancer and autoimmune disease [3]. In cancer, the expansion and activation of MDSCs are regulated by growth factors and inflammatory cytokines, which are released from tumor cells, tumor stromal cells, and immune cells. For example, tumor-derived cytokines, including IL-6 and IL-1β, as well as cytokines released from activated T cells, such as IL-4 and IL-10, develop common myeloid progenitor cells into MDSCs [26]. Hypoxia is another important activator of MDSCs; it increases the expression of HIF-1α in cancer cells [27]. Several inflammatory cytokines, growth factors, and HIF-1α start to be released from microglia and macrophages 3–6 hours after ischemia [28–31]; this is earlier than PMN-MDSC-LCs are recruited, and these factors may indeed be activators of the cells. Because hypoxia and these inflammatory mediators are one of crucial factors to introduce MDSCs [8], they might also play critical roles in inducing MDSCs in the early stage of the ischemic stroke [1]. The function of MDSCs in cancer is inhibiting T cell function, that exacerbate cancer [32], but MDSCs might alleviate ischemic injury by suppression of detrimental responses of γδ T cells, CD4^+^ T cells, and CD8^+^ T cells, which contribute to ischemic injury [33, 34]. To achieve such a strategy, transplantation of PMN-MDSC or administration of agents (for example, CCL2 [35]), which could positively act on PMN-MDSCs, should be examined in the future study.

One limitation of this study was that we could not examine any functional assays to confirm that the cells were PMN-MDSCs. We followed the algorithm of Bronte et al. [3], which recommends evaluating T cell inhibition using an enzyme-linked immunospot assay or T cell proliferation assay. However, we could not carry out such assays because we could not culture sorted cells from the ischemic brains. For this reason, we identified the cells as PMN-MDSC-LCs.

In summary, ischemic stroke increased the count of PMN-MDSC-LCs in the bone marrow, spleen, and ischemic hemisphere. Further studies involving PMN-MDSCs might clarify how the T cell immune response is regulated after ischemic stroke.

## Supporting information

S1 Table Statistics in Fig 2A. (PDF)

S2 Table Statistics in Fig 2B. (PDF)

S3 Table Statistics in Fig 2C. (PDF)

S4 Table Data in Fig 3. (PDF)

S5 Table Statistics in Fig 4. (PDF)
