# Supplementary material for: Temporal and spatial profile of polymorphonuclear myeloid-derived suppressor cells (PMN-MDSCs) in ischemic stroke in mice
Source: PLoS One. 2019 May 2;14(5):e0215482. doi: 10.1371/journal.pone.0215482 (PMC6497247; doi:10.1371/journal.pone.0215482)
Supplement: S1 Table — (PDF) [file pone.0215482.s001.pdf]

Supporting Information

S1 Table. Statistics in Fig 2A.

Normal (%)	Contralateral hemispere (%)			Ischemic hemispere (%)		
	24 h	72 h	120 h	24 h	72 h	120 h
0.093844	0.244145	0.4932426	0.064788	0.4533761	2.293909	6.885998
0.077782	0.3229011	0.5669318	0.528275	0.2242871	9.551449	0.881394
0.28877	0.0702206	0.4957858	0.34292	0.2958302	3.784687	1.636255
1.059071	0.1321179	0.6379418	0.497672	0.6137766	2.888817	2.147149
0.21815	0.1238053		0.663413	0.4790285	2.577159	6.034697
0.0916						

Kruskal–Wallis test	
P value	0.0004
Number of groups	7

Dunn's post hoc test	P value
Normal vs. contralateral hemisphere 24 h	ns
Normal vs. contralateral hemisphere 72 h	ns
Normal vs. contrarateral hemisphere 120h	ns
Normal vs. ischemic hemisphere24 h	ns
**Normal vs. ischemic hemisphere72 h**	**p < 0.01**
**Normal vs. ischemic hemisphere120 h**	**p < 0.05**
