# Supplementary material for: Temporal and spatial profile of polymorphonuclear myeloid-derived suppressor cells (PMN-MDSCs) in ischemic stroke in mice
Source: PLoS One. 2019 May 2;14(5):e0215482. doi: 10.1371/journal.pone.0215482 (PMC6497247; doi:10.1371/journal.pone.0215482)
Supplement: S2 Table — (PDF) [file pone.0215482.s002.pdf]

S2 Table. Statistics in Fig 2B

Normal (%)	24 h (%)	72 h (%)	120 h (%)
0.029646	0.206166	0.358833	0.026342
0.014267	1.669584	0.055335	0.104961
0.09542	0.492221	1.083166	0.996656

ANOVA summary	
P value	0.1342
P value summary	ns
Are differences among means statistically significant? (P < 0.05)	No
