# Supplementary material for: Temporal and spatial profile of polymorphonuclear myeloid-derived suppressor cells (PMN-MDSCs) in ischemic stroke in mice
Source: PLoS One. 2019 May 2;14(5):e0215482. doi: 10.1371/journal.pone.0215482 (PMC6497247; doi:10.1371/journal.pone.0215482)
Supplement: S3 Table — (PDF) [file pone.0215482.s003.pdf]

S3 Table. Statistics in Fig 2C

Normal (%)	24 h (%)	72 h (%)	120 h (%)
22.94082	21.04884	39.9583	58.27102
28.84731	19.70085	25.65719	39.22144
1.857828	15.33984	21.36766	20.19035
	2.803353		

ANOVA summary	
P value	0.4537
P value summary	ns
Are differences among means statistically significant? (P < 0.05)	No
