# Supplementary material for: Temporal and spatial profile of polymorphonuclear myeloid-derived suppressor cells (PMN-MDSCs) in ischemic stroke in mice
Source: PLoS One. 2019 May 2;14(5):e0215482. doi: 10.1371/journal.pone.0215482 (PMC6497247; doi:10.1371/journal.pone.0215482)
Supplement: S4 Table — (PDF) [file pone.0215482.s004.pdf]

S4 Table. Data in Fig 3

Gene	Ischemic hemisphere (mean fold change)	Contralateral hemisphere (mean fold change)	Spleen (mean fold change)
Nox2	1.9500	10.7875	2.1995
	2.5971	7.0511	1.1294
CHOP	6.6869	9.2618	4.7910
	17.2899	22.4046	13.9673
Nox4	0	0	0
	0	0	0
