# Supplementary material for: Temporal and spatial profile of polymorphonuclear myeloid-derived suppressor cells (PMN-MDSCs) in ischemic stroke in mice
Source: PLoS One. 2019 May 2;14(5):e0215482. doi: 10.1371/journal.pone.0215482 (PMC6497247; doi:10.1371/journal.pone.0215482)
Supplement: S5 Table — (PDF) [file pone.0215482.s005.pdf]

S5 Table. Statistics in Fig 4

section number	1	2	3	4	5
a	50	66	63	26	65
b	65	37	13	5	48
c	40	55	23	3	19

ANOVA summary	
P value	0.1117
P value summary	ns
Are differences among means statistically significant? (P < 0.05)	No
